# Improve Communicating Uncertainty in Intensive Care Unit With Patient and Family (ICU-PF)

**DOI:** 10.7759/cureus.20837

**Published:** 2021-12-30

**Authors:** Anhar Alhussaini

**Affiliations:** 1 Safety, Quality, Informatics and Leadership, Harvard Medical School, Boston, USA; 2 Health Professions, Johns Hopkins University, Baltimore, USA

**Keywords:** quality improvement projects, intensive care unit, patient-family centered care, patient-centered communication, uncertainty

## Abstract

Communicating uncertainty with patients and families in the intensive care unit is challenging and requires time and skill to convey the information. This proposal aims to provide a structured path for identifying and communicating uncertainty with patients and families in the unit. The focus is to improve the quality of care and timely communication to meet the expectations and needs of families and patients. The project focuses on the first 24 hours of intensive care unit admission to improve communication of uncertainty. By utilizing the Plan-Do-Study-Act cycle, the workflow uses a screening tool to identify uncertainty and communicate using evidence-based recommendations and the mnemonic VALUE (Value family statements, Acknowledge emotions, Listen, Understand the patient as a person, Elicit questions) as the standard of care. The workflow can be incorporated during the routine rounds as part of the A-F liberation bundle. The outcome is to improve patient and family satisfaction scores using a validated Family Satisfaction with Care in the Intensive Care Unit (FS-ICU 24) questionnaire to achieve a score of 75 or more, which correlates with very good. Challenges and limitations are discussed in the proposal.

## Introduction

Communicating uncertainty in the intensive care unit (ICU) to patients and families is challenging given multiple factors, including lack/limited information, complexity, and time pressure to manage sick and unstable patients [1]. This project aims to improve communication of uncertainty with patients and families in the ICU to enhance the quality of care and patient safety by being honest and transparent and disclosing information in a timely manner to build up trust with the healthcare team.

The project will utilize informatics to facilitate communication and share information. Leadership is a key in "times of uncertainty." This project will add value to patients' advocacy and trust buildup with the healthcare team by ensuring proper communication from the healthcare team to meet the expectations and needs of families and patients [2].

Major and minor focuses

The major focus is to improve the quality of care by improving communication during uncertain times, as defined by the first 24 hours of ICU admission.

Minor focus is to use informatics to enhance communication and improve patient safety by enhancing better and timely communication with the family.

## Technical report

Problem statement

There are uncertainties surrounding diagnosis, treatment, and prognosis in ICU. Patients and families usually look for more information to keep them informed, bring disclosure, and reflect the high care quality they received [2]. Often, communicating prognostic uncertainty in ICU can be misleading or confusing to patients and their family members [2]. In an interview conducted with 90 family members of patients who died regarding "communicating prognostic uncertainty," less than half of the family members were not satisfied with communicating the patient's likely outcome [2].

The project's scope will be patients admitted to ICU in the first 24 hours to improve the communication of uncertainty between the ICU team and patients and family members, assess response, develop an action plan, and provide support. Follow up and update when clinical status changes or new information is available. The project will not address uncertainty measurement, risk assessment, and evaluating how physicians respond and deal with uncertainty.

Furthermore, effective communication has been shown to improve satisfaction and end-of-life care experience and is associated with high-quality care from patient and family perspectives [2]. The project will help improve the quality of care delivered in ICU to patients and their families by improving communication and support through decision-making under uncertainty and building trust.

Learning community

The stakeholders are patient and family members, ICU team, nursing manager, nursing educator, nursing staff, allied health, social worker, and pharmacist.

Sponsor: Chief of ICU.

Team leader: Physician lead.

Project team: ICU physician, nursing manager, and social worker.

Aim statement

To improve communication of uncertainty in ICU with patients and families in the first 24 hours of admission by 75% within one year using quality metrics indicators.

SMART Aim

Specific: Focusing on improving communication uncertainty in the ICU with patients and families in the first 24 hours of ICU admission. The focus is "family-centered care" to improve the quality and experience of families in ICU by providing support [3].

Measurable: By measuring timely communication and satisfaction of the patient and family experience using the Family Satisfaction with Care in the Intensive Care Unit (FS-ICU 24) questionnaire, which has been validated, and one of its components is communication with the ICU team [4,5]. The questionnaire correlates with the quality of care [4]. FS-ICU 24 is self-administered and takes 10-15 minutes to complete [4]. The aim is to achieve at least a score of 75 or more, which correlates with very good [4]. By collecting feedback from physicians and nurses, we get insight into what works and the challenges of communicating uncertainty.

Attainable: Communication with family is part of daily encounters in ICU with the ICU multidisciplinary team. Most of the communication happens by physicians and bedside nurses. A total of 87% of surrogate decision-makers in semi-structured interviews of 179 surrogates to patients in ICU with a high risk of mortality wanted prognostic uncertainty to be discussed by physicians, acknowledging "uncertainty is inevitable" [6]. Finding, along with the study by Krawczyk and Gallagher (2016), shows that families and surrogate decision-makers appreciate open and honest communication [6].

Relevant: Communication with family is part of the A-F ICU liberation bundle ("A: Assess, prevent, and manage pain; B: Both spontaneous awakening trials and spontaneous breathing trials; C: Choice of analgesia and sedation; D: Delirium: assess, prevent, and manage; E: Early mobility and exercise; F: Family engagement and empowerment"), which is recommended by the Society of Critical Care Medicine and used by ICU teams daily during rounds [7]. The bundle has been shown to improve outcomes in ICU, and the F component refers to family engagement and empowerment [8]. It is also an essential part of "shared decision-making" in the ICU to establish patients' values and care goals [8,9]. It aligns with the concept of "family-centered care," which is defined as "an approach to health care that is respectful of and responsive to individual families' needs and values" [3]. As from surrogate decision-makers and families, the relevance of discussing uncertainty from their perspective is to allow them to be prepared mentally for the worse outcome, hope for the best, make decisions, and promote trust with the ICU team [6].

Time-bound: The project will run for 12 months, with a monthly review of both the FS-ICU24 questionnaire and feedback from the staff to adjust or make a change.

Proposed intervention

**Figure 1:**
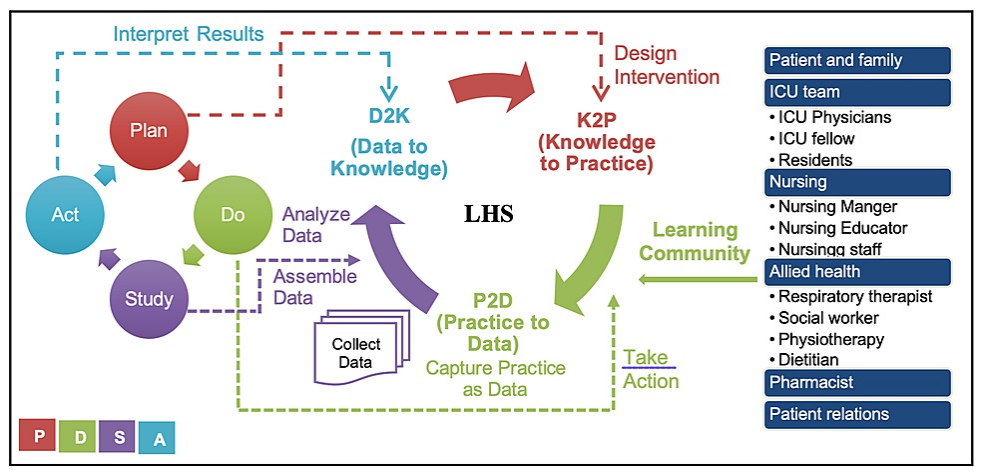
Learning health system, learning community, and PDSA cycle PDSA: Plan, Do, Study, and Act; LHS: learning health system.

**Figure 2:**
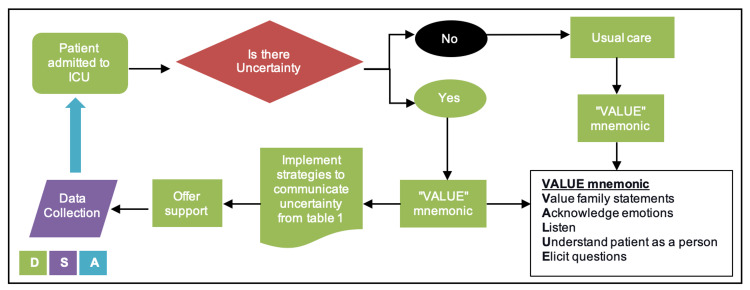
Workflow for communicating uncertainty with families in the ICU

Following the learning health system in data to knowledge (D2K) phase (Figure 1), identify a need to improve communication with family members of critically ill patients in the ICU, especially in the coronavirus disease 2019 (COVID-19) pandemic, where challenges include remote communication, more uncertainties resulting in anxiety and fear. Our focus is mainly on quality improvement using PDSA (Plan, Do, Study, and Act) cycle for rapid testing and implementing changes. The project starts by planning a workflow (Figure 2) and communication tools to be used by the ICU team that follows a structured approach of communicating uncertainty from evidence-based recommendations (Table 1) [10]. The mnemonic VALUE (Value family statements, Acknowledge emotions, Listen, Understand the patient as a person, Elicit questions) will be the usual care of communication [3].

**Table 1:** Strategies to communicate uncertainty Adopted from Simpkin and Armstrong (2019) [10]. CBT, cognitive behavioral therapy.

Domain	Strategies
Explicitly assess patients' desire for information and method of delivery for that information	Assess an individual's informational preferences and capacity for understanding uncertainty.
	Tailor conversation for individual, altering specific type/amount of information according to various characteristics (gender, culture, education, psychological factors, behaviors of interest) that relate to patients' capacity to use/respond to such information.
Strategies to communicate risk and ambiguity	Bracket estimates with ranges to convey realistic uncertainty, being sure to allow for exceptions in both optimistic and pessimistic directions.
	Round off numbers to avoid false illusions of precision.
	Use qualitative descriptions but beware that many have no generally accepted anchoring at specific quantitative levels of frequency; may work to relate medical risks to nonmedical risks so they can be placed in a larger perspective of a persons' life.
	Visual aids to communicate probabilistic information improve cognitive outcomes.
	Be aware of framing effects in conveying information on uncertainty which may impact uncertainty aversion: for example, gains versus losses; qualitative versus quantitative.
	Consider presenting risk information in several formats (qualitative, graphical displays, positive frame, negative frame, frequency, proportions, absolute, relative) to avoid framing biases in the perception of the message.
Ensure support is fostered	Education/communication approach: CBT to improve patients' resilience and ability to cope with uncertainty.
	Clarify the type of uncertainty that is most distressing to the patient and explain the complexities of each: uncertainty about probabilities; uncertainty about sources of information; uncertainty about evidence.
	See uncertainty as an opportunity rather than danger.
	Provide emotional support: "With you on the journey"; "I do not know, but I will be there no matter what happens" takes humility and a commitment to a meaningful engagement, that commitment is often what patients want most.
	Assure will answer all questions, provide resources, inform of own biases and values, inform of alternative treatments.
Clarify plan	Safety-netting is often used especially if a diagnosis is uncertain, and differential includes serious illness: say precisely what to look for; say precisely how to seek further care; be precise about time course.

The time of administration will be with the first encounter and on every subsequent meeting with family members. These time points can be during a scheduled family meeting or when given an update. Family members are offered support during the process. The support can be provided by the ICU team, nursing staff, social workers, and spiritual care. The response and feedback from patients, family members, and providers will be collected. Studying monthly reviews of responses and feedback to identify any changes to the process or modifications to the workflow.

By structuring evidence-based communication tools and workflow to support family members during their ICU experience, we can improve communicating uncertainty to family members of critically ill patients in ICU and identify and provide support to those who need it. The timeline of PDSA will be as follows: four months for a plan, 12 months for do, and monthly for study and act. The FS-ICU 24 can be accessed online [5].

Measurement

The primary outcome is patients and family satisfaction with ICU experience to achieve a score of 75 or more, which correlates with very good [4]. Secondary outcomes will measure process and structure metrics, as shown in Table 2.

**Table 2:** Measurement

Types of measure	Metrics	Data source	Baseline	Performance target
Outcome	Satisfaction in the overall care	Survey	Unknown	Score 75 [4]
	Satisfaction in decision-making	Survey	Unknown	Score 75 [4]
	Total family satisfaction	Survey	Unknown	Score 75 [4]
Process	Cycle time (the average time from patient admission to ICU to family meeting)	Chart review	72 hours [11]	24 hours
	Average meeting time spent with family	Chart review	30 minutes [12]	Same or less
	The number of times supports initiated, including the number of referrals to spiritual care and social worker	Chart review	Unknown	More
Structure	Staffing of the ICU day vs. night	Admin data	Variable	Less variable
	Support structures need after hours and over the weekend	Admin data	Unknown	Establish

**Figure 3:**
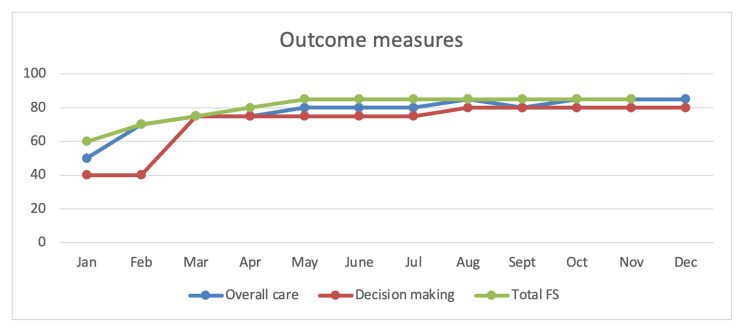
FS-ICU 24 over 12 months projection FS-ICU: Family Satisfaction with Care in the Intensive Care Unit; FS: family satisfaction.

The data review will be done monthly by the project team to assess the ongoing need for adjustment and improvement (Figure 3). The data review ties to the study and action phases of the PDSA cycle. A baseline data collection will be done during the planning phase for comparison.

Challenges

We need to anticipate multiple challenges and barriers; the most important one is related to the COVID-19 pandemic and the limitation of resources. Patients admitted to ICU with COVID-19 add another layer of complexity to the uncertainty and the fear and anxiety associated with it. The following are the challenges and suggested strategies [13-15]:

1. Staff training: This will require staff training to deliver effective communication, documentation of encounters, and duration. It can be done through learning modules, printed cards, guideline documents, and structured notes.

2. COVID-19 pandemic: Since the COVID-19 pandemic started, a lot of innovations have been tried and shared. Those innovations are mainly surrounding the delivery and the communication of care. Due to restrictions on hospital visits, virtual visits have been offered as a way of communication. Of course, the challenges are usually in familiarity with technology, malfunction, lack of face-to-face interaction, and loss in translation. This can be made better by having infrastructure, a reliable and secure platform, and allocating designated person to communicate.

3. Time challenges: Due to the nature of ICU, time can be challenging to meet family members, and usually, meetings are scheduled. With the restricted visiting hours, families are not by the bedside as at times of pre-COVID-19 pandemic, and this puts some pressure on the ICU team to update families after their daily rounds. Suggest identifying team members responsible for communicating updates with family members to help be consistent, build rapport, and take off some of the team's pressure.

4. Resources limitation: These include limited access to the social worker for every patient and family member. Propose identifying those who will likely require referral support.

5. Process: There are opportunities to improve communication in two situations:

(a) Transfer from ICU: Communicating with family members upon transferring patients out of ICU is important. Those usually are emotional times for fear of returning to ICU, break-in care, and what is next [14]. The assurance from the ICU team on communicating the transfer to family members should also include the follow-up by the rapid assessment of critical events (RACE) team.

(b) End-of-life care/palliative care: These conversations require skillful communication to ensure discussions of goals of care are met and the family feels supported during the process.

6. Structure: This includes the following:

(a) Culture: There might be some reservations to acknowledge uncertainty openly, and a culture of "uncertainty is inevitable" is encouraged to help facilitate those types of discussions. Some patients and families from specific backgrounds do not like to know. Thus, in the recommendations in Table 1, explicitly assessing the desire to know the information is the first step to establishing their perspective.

(b) Incentive: Hospitals might want to improve communications with family members in the ICU by hiring more support personnel and investing in continuous improvement projects to improve the process.

(c) Champions: Along the way, it helps to identify champions from the ICU team and family members as advocates to support others.

7. Acknowledgment and trust: Once the ICU team and family acknowledge uncertainty, trust can be built toward a common goal that both are working toward achieving. Communication is a vital part of the trust framework that is suggested by van der Voort and Lemmers in a recent review [15]. The downside of acknowledging uncertainty is confusion by the family members. This can be avoided by explicitly mentioning the steps taken to investigate and manage and the timeline expected. Assigning one person to communicate with from the ICU team and a spokesperson from the family help avoid misinformation and mixed messages and lessen the confusion.

Sustainability

The project improves everyday communication with family members in the ICU to lessen the uncertainty and provide support. The project can be easily incorporated into the daily A-F ICU liberation bundle, the first encounter with family, every encounter, and scheduled meetings.

Working with the information technology (IT) department to design a form integrated into the electronic patient record (EPR) can help the ICU team conduct and document the meeting. Integrating the project into residents' and fellows' teaching will expand and ensure sustainability through training and EPR documentation.

Given that communication is a crucial part of the trust framework, and perceived quality of care is linked to patient and family satisfaction, leadership from the hospital and family members will ensure sustainability and provide the support needed to maintain the structure. These include hiring additional supporting personnel, incentive, and investing in a reliable and secure platform to conduct the virtual meetings.

Continue to measure FS-ICU 24, the cycle time to improve early communication, and support needed. To convey the data, use the ICU board to display FS-ICU-24 results monthly.

Scalability

For scalability, looking at five elements:

1. Effectiveness: Given the importance of improving communication with patients and families, especially in critical care in times of uncertainty, the project can expand to other areas that deal with uncertainty, including the emergency department, primary care, internal medicine, and surgical departments. The concept of communicating uncertainty starts after establishing rapport with the family using either the VALUE mnemonics or other communication skills methods and then using the proposed recommendations suggested by Simpkin and Armstrong (2019) [10].

2. Education and training are key to performing communication skills. This will require an added session to the ICU fellowship curriculum and to rotating ICU residents to discuss how to communicate uncertainty. Staff training and online module will help with sustainability.

3. Resources: Need training and infrastructure to support additional resources for family members, including support personnel, volunteers, and hiring an additional social worker.

4. Cost: Might need to hire support personnel trained to provide emotional support as required.

5. System: The crucial part is the support of families when dealing with uncertainty and giving thoughtful consideration to putting a support system in place that they can access 24/7 and partner with the community for rapid access.

The plan for dissemination will involve writing up our ICU experience for critical care centers to implement. The topic could be discussed in a formal interdepartmental ground round with internal medicine and the emergency department on a hospital level.

## Discussion

It is crucial to communicate clearly with the patients and families during critical care admission [2]. The uncertainty surrounding the diagnosis and prognosis in the ICU causes many miscommunications and can lead to misleading information if not communicated correctly [2]. Therefore, it is important to establish a communication tool for these scenarios to avoid the consequences of miscommunications. While keeping an open channel is vital, having assigned members who communicate with family establishes rapport and trust-building [3]. This is done as part of the A-F bundle during daily ICU rounds as well as whenever there is a change of status [7]. This encapsulates the patient and family-centered care model in ICU by improving the communication with its members [2,8].

During the COVID-19 pandemic, the use of technology has increased to allow for social distance [16]. This has taken the form of using video calls with family, which has been incorporated in ICUs to enable families to attend rounds virtually and communicate with the ICU team [17]. Offering alternative methods of communication will need to be reliable and secure. When possible, incorporating informatics to guide and document communication with families is an integral part of the patient medical record. This will also help track the timing and frequency of communication.

The proposal will have an impact on safety and quality by allowing for open and honest communication and keeping the patient at the center of care. This will also empower families and provide needed support to help with care during ICU admission and transition of care. As discussed above, challenges and limitations are important to identify and address. The project will need a continuous iteration of the PDSA cycle to continue incorporating feedback.

## Conclusions

The project presents a unique structured tool in communicating uncertainty during the ICU stay. This will positively impact safety and quality by engaging patients and families with the ICU team. Using a structured communication tool helps with conveying the information while allowing for dialogue and discussion. Future research studies are needed to address the gap in communicating uncertainty in ICU.
